# Genetically integrated traits and rugged adaptive landscapes in digital organisms

**DOI:** 10.1186/s12862-015-0361-x

**Published:** 2015-05-12

**Authors:** Elizabeth A Ostrowski, Charles Ofria, Richard E Lenski

**Affiliations:** Department of Biology and Biochemistry, University of Houston, Houston, TX 77204 USA; Department of Computer Science and Engineering, Michigan State University, East Lansing, MI 48824 USA; BEACON Center for the Study of Evolution in Action, Michigan State University, East Lansing, MI 48824 USA; Department of Microbiology and Molecular Genetics, Michigan State University, East Lansing, MI 48824 USA

**Keywords:** Pleiotropy, Epistasis, Genetic correlations, Digital evolution, Experimental evolution

## Abstract

**Background:**

When overlapping sets of genes encode multiple traits, those traits may not be able to evolve independently, resulting in constraints on adaptation. We examined the evolution of genetically integrated traits in digital organisms—self-replicating computer programs that mutate, compete, adapt, and evolve in a virtual world. We assessed whether overlap in the encoding of two traits – here, the ability to perform different logic functions – constrained adaptation. We also examined whether strong opposing selection could separate otherwise entangled traits, allowing them to be independently optimized.

**Results:**

Correlated responses were often asymmetric. That is, selection to increase one function produced a correlated response in the other function, while selection to increase the second function caused a complete loss of the ability to perform the first function. Nevertheless, most pairs of genetically integrated traits could be successfully disentangled when opposing selection was applied to break them apart. In an interesting exception to this pattern, the logic function AND evolved counter to its optimum in some populations owing to selection on the EQU function. Moreover, the EQU function showed the strongest response to selection only after it was disentangled from AND, such that the ability to perform AND was lost. Subsequent analyses indicated that selection against AND had altered the local adaptive landscape such that populations could cross what would otherwise have been an adaptive valley and thereby reach a higher fitness peak.

**Conclusions:**

Correlated responses to selection can sometimes constrain adaptation. However, in our study, even strongly overlapping genes were usually insufficient to impose long-lasting constraints, given the input of new mutations that fueled selective responses. We also showed that detailed information about the adaptive landscape was useful for predicting the outcome of selection on correlated traits. Finally, our results illustrate the richness of evolutionary dynamics in digital systems and highlight their utility for studying processes thought to be important in biological systems, but which are difficult to investigate in those systems.

## Background

Pleiotropy and epistasis lie at the heart of much of evolutionary theory. Together they give rise to the complex mapping between genotype and phenotype, and they can generate constraints on adaptation. Pleiotropy is a major cause of genetic correlations, which can hinder the response to selection on one trait owing to its association with another [1,2]. Pleiotropy can thus prevent traits from being independently optimized. However, even where pleiotropy does not present an absolute constraint (e.g., compensatory evolution may alleviate a pleiotropic trade-off), it can still prolong an approach to the optimum and lead to temporarily maladapted states [1-6].

Epistasis can also constrain evolutionary outcomes by pushing evolution along some paths and away from others [1,2,7]. In particular, reciprocal sign-epistasis for fitness is necessary (but not sufficient) to generate rugged adaptive landscapes, with the potential to trap populations on local fitness peaks that are globally suboptimal [8-10]. Despite the conceptual appeal of envisioning adaptation as a process that unfolds on rugged adaptive landscapes, it is generally difficult to observe that process [11,12]. Even when there is evidence that populations have reached alternative peaks [13,14], it can be difficult to determine key attributes of the adaptive landscape. For example, hybrids of low fitness could indicate an intervening valley, but whether the evolutionary trajectory actually traversed the valley is typically unknown. An alternative possibility is that the populations diverged around a ridge, such that the true adaptive landscape is shaped like the cone of a volcano rather than comprised of two peaks with an unavoidable valley between them [15,16]. Thus, understanding the process of evolution on adaptive landscapes requires detailed knowledge of both the trajectory of the evolving populations and the fitness effects of the contributing mutations, but acquiring these data is not feasible in most systems.

Avida is a software platform for performing experimental studies on evolving systems in a computational setting. In Avida, self-replicating computer programs mutate, compete, and evolve in an open-ended manner [17-28]. Several features of Avida make it well suited for addressing questions in the realm of evolutionary genetics. First, the speed of the computational process makes it feasible to examine both short- and long-term evolutionary dynamics. Second, individuals or entire populations can be saved and restored at a later time, permitting direct comparisons of evolved and ancestral genotypes. Third, it is possible to describe the exact trajectory of all evolving populations and to determine the fitness effects of all mutations along the line of descent leading from the ancestor of an experiment to an evolved organism or population. Fourth, a powerful set of genetic tools and tests are available, including one that generates the complete one-step (or even multi-step) mutational neighborhood of any genotype of interest. Fifth, with 26 different instructions possible at any site, and genome sizes ranging from tens to thousands of instructions, the number of possible genotypes in the system is vast, easily exceeding the number of atoms in the universe. It is thus possible to study evolution in genetically diverse populations that are potentially far removed from a state of equilibrium. Sixth, perhaps the most important attribute of Avida is the complex, nonlinear mapping between genotype and phenotype, a property that leads to the emergence of pleiotropy and epistasis. These last two qualities differentiate digital evolution from many other models or simulations of the evolutionary process. Because of extensive pleiotropy and epistasis, evolution in Avida potentially involves limitations and constraints on adaptation that are, at least conceptually, the same as those that limit biological evolution.

In this paper, we describe a set of experiments to examine the evolutionary response of genetically coupled functional traits in digital organisms. When multiple traits share an underlying genetic basis, they may not evolve independently, resulting in constraints on adaptation or even maladaptation [1,29-31]. Artificial selection experiments can be useful for examining constraints [30,32], and some studies have shown that genetically correlated traits can be uncoupled through artificial selection [33-36]. Nevertheless, little is known about how genetically coupled traits influence long-term outcomes [3]. For example, theory suggests that genetic correlations are unlikely to constrain evolution permanently in an environment with only a single optimum [1]. However, genetic correlations can be important in directing evolution toward a particular adaptive peak in a multi-peaked environment, and thus for directing evolutionary outcomes over longer times scales [37-39].

**Figure 1:**
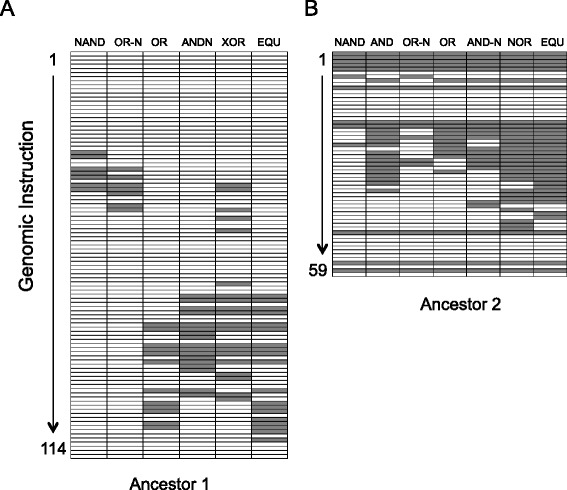
Genotype-phenotype maps for the ancestral organisms used in this study. Each row represents one instruction, starting from the first instruction in the organism’s genome (top row) to the final instruction (bottom row). Each column represents one of the logic functions performed by the ancestor. The color of each cell indicates what happens to the performance of a function when the instruction in that row is knocked out (replaced with a null instruction). White: knocking out the instruction does not affect performance of the function. Gray: knocking out the instruction causes the function to be lost. **(A)** Map for Ancestor1. Note that every instruction that knocks out OR also knocks out EQU, but the reciprocal is not true. **(B)** Map for Ancestor2. Note that every instruction that knocks out either AND or OR also knocks out EQU.

In digital organisms, the relevant traits are logic functions, i.e., computations that these organisms may evolve the ability to perform using numbers they input from their environment. Computation of logic functions provides the organisms with additional energy, which they can use to reproduce. The specific computations that we examine here were identified in a previous study in which replicate populations evolved in an environment where only a single computation, the logic function EQU, was directly selected [19]. A major result of that work was that selection to increase outputs of the EQU computation often led to a correlated increase in the outputs of other, unselected computations. Moreover, the reason for the correlated increases became apparent when we examined the way in which different computations were encoded in the genome. Specifically, functions prone to correlated increases exhibited high degrees of genetic overlap with the selected EQU function (Figure 1). This result indicated that pleiotropy—in the sense that the same portions of the genome determined the expression of multiple logic functions—was the cause of their correlated evolution. However, because only one trait was under selection, it was impossible to say whether the independent evolution of the logic functions would be constrained in any way by their genetic association.

Here we examine whether these functions are capable of evolving independently in a variety of environments that differ in the extent and direction of selection on each function. For each pair of functions, we examined their evolution in two types of environment; in the first case only one function was selected to increase (and the other evolved only as a correlated response to selection on the first), whereas in the second case one function was selected to increase while the other was selected to decrease. We show that correlated responses, while often asymmetric, could usually be broken when selection was applied in opposing directions on the two traits. In an interesting exception, two traits evolved coordinately despite selection in opposing directions, such that one trait evolved counter to its phenotypic optimum. We take advantage of the transparency of the Avida system to explore this result, and we examine how the ruggedness of the adaptive landscape influences the evolution of these two traits.

## Methods

### The Avida system

Avida is a research platform wherein computer programs—called digital organisms—self-replicate, mutate, compete, and evolve [20]. Each digital organism consists of a genome (a sequence of computer instructions) and its associated virtual hardware, including stacks and registers used for storing and manipulating numbers, respectively. The genome of a digital organism contains information for copying itself, such that execution of the instructions contained in the genome by a virtual CPU results in the production of an offspring program. The copy process is subject to random mutations, which provide the variation required for natural selection to occur. Mutations can be substitutions, whereby an instruction in the parent genome is randomly replaced with a different one in the offspring. Alternatively, they can be insertions or deletions, whereby extra instructions are added or lost, respectively. As in natural systems, some of these mutations are neutral, in that they do not affect the organism’s fitness. Most other mutations are deleterious or lethal, because they either reduce the speed with which an organism produces copies of itself or destroy that ability altogether. A reduction in speed can take many forms, such as a reduced ability to acquire resources, a less efficient copying algorithm, or an increase in the amount of genetic material to be copied. Occasionally, though, mutations are beneficial and result in higher rates of reproduction, and those mutants will tend to increase in frequency in the population over time.

Digital organisms use virtual CPUs to execute the instructions in their genomes and thereby replicate. CPU cycles are thus akin to energy—those organisms that acquire more CPU cycles from the environment process their genomes faster than those with fewer CPU cycles. All else being equal, faster processing should result in a higher rate of reproduction. Selection can also favor organisms that use CPU cycles more efficiently, for example, by reducing the number of CPU cycles required to produce an offspring. Alternatively, organisms can use CPU cycles to perform computations, which can, in turn, provide them with additional CPU cycles. These computations are logic functions that the organisms perform on binary numbers (strings of 0 s and 1 s) input from their environment as a form of digital metabolism. The logic functions are as follows: NOT, NAND, AND, OR, OR_N, AND_N, NOR, XOR, EQU. They are described in greater detail elsewhere [22]. For the purposes of the current study, *rewarded* functions yield additional CPU cycles to those organisms that perform them, whereas *punished* functions cause CPU cycles to be taken away.

Genomes may contain instructions that dynamically alter the position in the genome currently being expressed. For example, digital organisms typically use a loop to copy their genomes instruction by instruction, exiting the loop when the copy is complete. They can also perform logic functions using loops and may even incorporate logic functions into their copy loops, which can generate correlations between genome length and the number of times a logic function is performed. Digital organisms often use the result of one computation as an input (or partial result) for another computation; this re-use helps explain the genetic overlap among different functions and the tendency for the performance of one function to be correlated with that of another (see also [19] and [22]). This genetic integration is not required – the organisms can calculate these functions separately, although it may be less efficient to do so. Moreover, there are multiple ways to build complex functions, such as EQU, out of simpler functions. Thus, the identity of the simpler functions that are integrated into the EQU function will often depend, in part, on which of the simpler functions evolved first.

### Experimental design

In a previous study, we examined how non-selected functions were lost when generalist organisms evolved to specialize on a single function, EQU, and we evaluated the relative roles of antagonistic pleiotropy versus mutation accumulation in causing these losses of function [19]. In the first stage of those experiments, we evolved a number of generalists that could perform a variety of logic operations, including EQU. These generalist organisms then served as ancestors in the second part of the experiment, where replicate populations evolved to specialize on the EQU function. Here we focus on two of these generalist ancestors, called Ancestor1 and Ancestor2 for simplicity in the present study (but labeled as Ancestor1 and Ancestor3, respectively, in our previous study [19]), because the replicate populations that evolved from these ancestors in the EQU-only environment always maintained the OR function, despite losing most other unrewarded functions. Not only did the organisms that evolved from these ancestors fail to lose the OR function, but their performance of OR often increased in a correlated fashion with increased performance of EQU, as measured across replicate populations (Table 1) [19]. Organisms evolved from Ancestor2 in an EQU-only environment also showed correlated increases in their performance of the AND function (Table 2), in addition to OR. For this reason, we also examined the relationship between these two functions and EQU in populations evolved from Ancestor2.

**Table 1:** **Number of times that nine different logic functions were performed during a digital organism’s lifetime**

**Pop.**	**NOT**	**NAND**	**AND**	**ORN**	**OR**	**ANDN**	**NOR**	**XOR**	**EQU**
1	0	0	0	0	**91**	0	0	0	**361**
2	0	0	0	1	**1**	0	0	0	**1**
3	1	0	0	0	**1**	0	0	0	**3**
4	0	0	0	0	**101**	0	0	0	**101**
5	0	0	0	0	**32**	0	2	0	**62**
6	0	0	0	0	**1**	0	0	0	**2**
7	0	0	0	0	**1**	0	0	0	**2**
8	0	0	0	0	**1**	0	0	0	**1**
9	0	0	0	0	**1**	0	0	0	**1**
10	0	0	0	0	**44**	0	0	0	**130**

Numbers are for the dominant genotypes from 10 replicate populations derived from Ancestor1 in the +EQU environment, where selection was for the EQU trait only. The correlation coefficient, *r*, between OR and EQU is 0.806. Data from [19].

**Table 2:** **Number of times nine different logic functions were performed during a digital organism’s lifetime**

**Pop.**	**NOT**	**NAND**	**AND**	**ORN**	**OR**	**ANDN**	**NOR**	**XOR**	**EQU**
1	1	117	**118**	117	**118**	0	0	0	**118**
2	0	0	**100**	0	**100**	0	0	0	**298**
3	1	140	**141**	140	**141**	141	0	0	**141**
4	1	0	**77**	0	**77**	0	0	0	**152**
5	104	0	**104**	0	**104**	0	0	0	**104**
6	1	164	**165**	164	**165**	0	0	0	**322**
7	209	0	**208**	0	**208**	0	0	0	**208**
8	1	0	**185**	0	**185**	185	0	0	**369**
9	123	1	**124**	1	**124**	124	0	0	**124**
10	0	114	**109**	0	**109**	0	0	0	**325**

Numbers are for the dominant genotypes from 10 replicate populations derived from Ancestor2 in the +EQU environment, where selection was for the EQU trait only. The correlation coefficient, *r*, between AND and EQU and between OR and EQU is 0.368 in both cases. Data from [19].

Each experiment tested the relationship between two functions that the ancestor was able to perform. Specifically, we evolved replicate populations founded with either Ancestor1 or Ancestor2 in each of four environments. In two environments, we rewarded the performance of only one of the functions, while neither punishing nor rewarding the other. In the other two environments, we still rewarded one function but actively punished the performance of the other function by reducing an organism’s CPU cycles. To name each environment, we use + or - to indicate whether a function was rewarded or punished, respectively. For example, in the case of EQU and OR, we evolved replicate populations in the following four environments: +EQU (rewards EQU), +EQU/-OR (rewards EQU and punishes OR), +OR (rewards OR), and +OR/-EQU (rewards OR and punishes EQU).

We replicated each evolution experiment 100 times, for a total of 1100 runs (3 pairs of functions × 4 environments × 100 replicates, except the same set of 100 +EQU runs for Ancestor2 was used for the analyses of both OR and AND). We ran all experiments for a period of 100,000 updates, which was equal to an average of 11,223 generations for Ancestor1 and 2,845 generations for Ancestor2. The difference in average number of generations reflects evolved differences in gestation time, with the populations that evolved from Ancestor2 having longer generations because they spend more time performing logic functions. The genomic mutation rate for all experiments was set to 0.1 mutations per genome per generation, which included point, insertion, and deletion mutations at rates of 0.08, 0.01, and 0.01, respectively. In addition to these explicitly defined mutations, digital organisms may also occasionally undergo *implicit* mutations that result in the deletion or duplication of multiple instructions. An implicit mutation typically follows an explicit mutation that causes the asymmetrical division of a copied genome, where one of the asymmetric copies can then faithfully and successfully self-replicate [22]. Population sizes were fixed at 3600 organisms, and each new offspring replaced a random organism in the population.

The number of CPU cycles that an organism receives per unit time (its metabolic rate) is determined by a value called *merit*. An organism’s *base merit* is proportional to its genome length, such that there is no inherent selection for the genomes to shrink or grow in length (see [40] for additional information). Rewards and penalties for performing the various logic functions increase or decrease this value, respectively. The magnitude of the rewards and penalties were based on the function’s complexity [22] and the number of times it was performed per reproductive cycle. The functions were weighted as follows: EQU at 5, OR at 3, and AND at 2. These weights were multiplied by the number of times an organism performed the function to determine a penalty or reward. For example, if an organism performed EQU three times in an environment where EQU was rewarded, it received 15 times as many CPU cycles as an otherwise equivalent organism that did not perform EQU. The multiplier could be positive or negative, however, depending on whether the function was rewarded or punished, respectively. Thus, if the same organism also performed the AND function three times, and if the environment penalized AND while rewarding EQU, then its net multiplier would be only 9 (=3 × 5 – 3 × 2) relative to an organism that performed neither EQU nor AND.

At the end of each experimental run, we isolated the numerically dominant genotype from each population and measured its performance of the various logic functions. For focal populations of interest, we also determined the line of descent—that is, the sequence of all genotypes from the ancestor to this final organism. We examined the line of descent to identify the mutations that led to the loss of a particular function, and we determined the fitness effect of each of these mutations in the environment in which they arose as well as other environments. To quantify the fitness effects, we placed each genotype on the line of descent in a test CPU and assayed its ability to self-replicate, the number of instructions that it executed to produce an offspring (its gestation time), and the number of times that it performed each logic function. In a few rare cases, the test CPU failed to assign a fitness value to a genotype, which can occur when organisms have extremely long gestation times that exceed the time limit of the test CPU. By comparing the fitness of a given genotype to that of its immediate predecessor on the line of descent, we could examine how varying the magnitude and direction of selection on particular functions altered the topology of the adaptive landscape and thereby influenced the trajectory of evolution.

## Results

### Direct and correlated responses to selection on functions OR and EQU in Ancestor1

**Figure 2:**
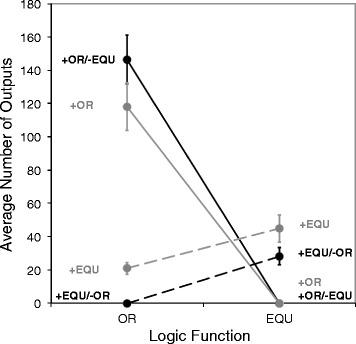
Average performance of functions OR and EQU by organisms evolved from Ancestor1. Ancestor1 performed each function only once per reproductive cycle. The y-axis shows the number of times a given logic function is output per reproductive cycle. Each point represents the mean value, based on 100 replicate populations in each environment, based on the most common genotype at the end of the experiment. The environment is shown next to each point, and lines connect measurements made on the same set of populations. Error bars indicate one standard error. All 100 populations lost OR in the +EQU/-OR environment, and all 100 populations lost EQU in both the +OR/-EQU and +OR environments.

Figure 2 shows the results of experiments to examine the evolutionary association between OR and EQU in Ancestor1. At the start of the experiment, the ancestor could perform each of these functions only once. Each point represents the average number of times a given function was performed by 100 independently evolved organisms, as a function of the environment in which they evolved. Starting on the left side of the figure, the data show that the performance of OR was higher in the two environments where it was rewarded, +OR and +OR/-EQU, than in the two environments where it was either not rewarded or punished (comparing upper left to lower left). This result indicates that OR responded positively to direct selection. Of the environments in which it was rewarded, the performance of OR was higher when EQU was punished than when it was not (mean = 146.07 in +OR/-EQU environment versus mean = 117.90 in +OR environment), although this difference was marginally non-significant (Mann–Whitney U = 5763, n = 200, *P* = 0.062). Focusing on the lower left part of the figure, the performance of OR also increased above the ancestral level of 1 as a correlated response to selection on EQU (mean OR = 21.1 in +EQU environment). However, when OR was punished, it was invariably lost (mean OR = 0 in +EQU/-OR environment). This result indicates that, despite the correlated response of OR to selection for EQU, the association between these functions could be broken when selection acted on them in opposing directions.

The response of EQU to these same environments is shown on the right in Figure 2. Its performance, like that of OR, evolved to higher levels in the environments where it was directly selected (i.e., the +EQU and +EQU/-OR environments). The performance of EQU did not differ significantly depending on whether OR was being punished or not (Mann–Whitney U = 4386, n = 200, *P* = 0.128), although EQU tended to be performed more often when OR was not punished. EQU, again like OR, was always lost when its performance was punished (mean EQU = 0 in the +OR/-EQU environment). However, EQU was also invariably lost when only OR was rewarded. These results thus reveal an asymmetry in the correlated responses: selection for EQU (+EQU environment) resulted in a correlated increase in the performance of OR, but selection for OR (+OR environment) led to the complete loss of EQU.

This asymmetry is not surprising in light of the genotype-phenotype map for Ancestor1 (Figure 1A). This map shows that all the genomic instructions that, when deleted, knock out the OR function also knock out the EQU function. However, the reverse is not true: some instructions can be deleted that knock out EQU without affecting the performance of OR. Put another way, the instructions encoding OR are a subset of those encoding EQU. The observed asymmetry in the correlated responses thus reflects the underlying asymmetry in the mapping between genotype and phenotype for these two traits.

### Direct and correlated responses to selection on functions OR and EQU in Ancestor2

**Figure 3:**
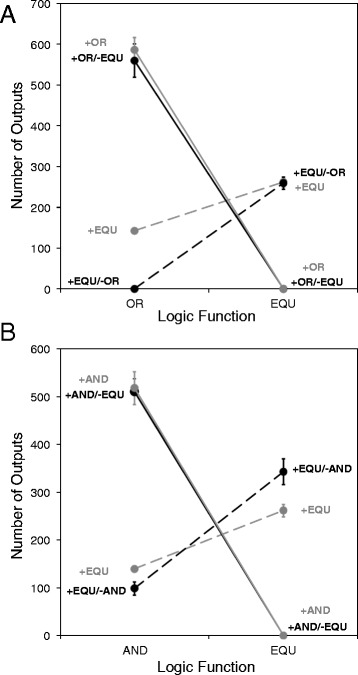
Average performance of functions OR, AND, and EQU by organisms evolved from Ancestor2. Ancestor2 performed each function only once per reproductive cycle. **(A)** Evolved performance of OR and EQU. **(B)** Evolved performance of AND and EQU. See the legend to Figure 2 for description of the points and error bars. All 100 populations lost OR in the +EQU/-OR environment, and all 100 populations lost EQU in both the +OR/-EQU and +OR environments. Also, all 100 populations lost EQU in both the +AND/-EQU and the +AND environments. However, only 47 populations lost AND in the +EQU/-AND environment.

Qualitatively similar patterns arose when we examined the correlation between OR and EQU in populations evolved from Ancestor2 (Figure 3A). This ancestor also performed OR and EQU only once. Selection for EQU (+EQU environment) led to a correlated increase in OR (lower left), but selection for OR (+OR environment) led to the complete loss of EQU (lower right). Once again, the direct response to selection was stronger than the correlated response to selection, with the performance of OR higher in the +OR and +OR/-EQU treatments than in the +EQU or +EQU/-OR environments (comparing upper left to lower left). Similarly, the performance of EQU was higher in the +EQU and +EQU/-OR treatments than in the +OR and +OR/-EQU environments (comparing upper right to lower right). In both cases, punishing performance of the alternative function had little effect on the evolution of the rewarded function; thus, the performance of OR did not differ appreciably between the +OR and +OR/-EQU treatments (upper left), and the performance of EQU was indistinguishable in the +EQU/-OR and +EQU treatments (upper right).

### Direct and correlated responses to selection on functions AND and EQU in Ancestor2

At first glance, the pattern was similar when we examined the functions AND and EQU in populations that evolved from Ancestor2 (Figure 3B). Selecting for the performance of EQU resulted in a correlated increase in the performance of AND from its ancestral level of 1 (lower left), but selecting for AND caused EQU to be invariably lost (lower right). This result was also expected from the genotype-phenotype map for this ancestor; all genomic instructions that, when deleted, knock out AND also knock out EQU, but the reverse is not true (Figure 1B). In other words, the genome instructions encoding the AND function are a subset of those encoding EQU.

However, the outcomes of these experiments differed from the preceding ones in two important respects. First, the performance of AND did not invariably decline to zero when it was selected against (Figure 3B, lower left). In fact, only 47 of 100 populations that evolved in the +EQU/-AND environment lost the ability to perform that function (Table 3; far right column). Second, EQU evolved to higher levels when AND was punished than when it was not (average performance of EQU = 342.8 in +EQU/-AND environment, compared to 261.2 in +EQU environment; Figure 3B, upper right), and this difference was significant (Mann–Whitney U = 5914.5, n = 200, *P* = 0.025).

**Table 3:** **Effect of punishment on loss of other functions**

	**Selected and other functions:**		**Other function was:**	
**Ancestor**	**Selected**	**Other**	**Not punished**	**Punished**
Ancestor1	EQU	OR	3	100
Ancestor1	OR	EQU	100	100
Ancestor2	EQU	OR	2	100
Ancestor2	OR	EQU	100	100
Ancestor2	EQU	AND	1	47
Ancestor2	AND	EQU	100	100

In all experiments, the “selected” function was rewarded, whereas the “other” function was not. “Not punished” and “punished” indicate the simultaneous direct selection, if any, on the other function. All experiments were replicated 100 times, and the numbers below indicate how often the other function was lost.

**Figure 4:**
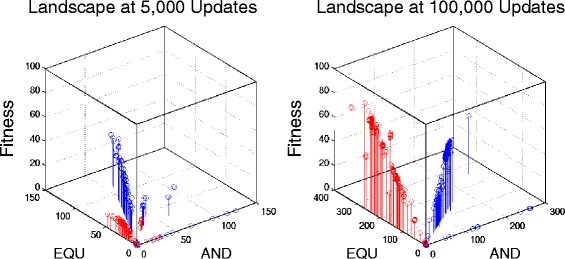
Snapshot of phenotypic variation present in two populations at two time points. Each point represents an individual organism; the height of the point shows its fitness in the +EQU environment, while the other two axes show the number of times it performs the EQU and AND functions. Blue: organisms from a population evolving in the +EQU environment. Red: organisms from a population evolving in the +EQU/-AND environment. Left: organisms that evolved in the +EQU/-AND environment (red) had lower fitness after 5,000 updates, when they were measured in the +EQU environment, compared to individuals that evolved in the +EQU environment (blue). Right: By the end of the experiment (100,000 updates), organisms that evolved in the +EQU/-AND environment (red) had attained higher fitness in the +EQU environment than those that evolved in the +EQU environment (blue).

One possible explanation is that the higher performance of EQU that evolved in the +EQU/-AND environment (relative to the +EQU environment) did not translate into higher overall fitness. For example, it may have caused a correlated increase in replication rate, such that the two outcomes—although different—represented equally good evolutionary outcomes. To see if these organisms did indeed have equivalent fitness, we took the ones evolved in the +EQU/-AND environment and transplanted them to the +EQU environment, where we assayed their fitness. Surprisingly, we found that they were also significantly more fit in that environment than the organisms that had evolved there (Mann–Whitney U = 6220, n = 200, *P* = 0.003). A similar pattern, where a population reaches higher fitness in one environment through evolution in some alternative environment, has been observed in laboratory-evolved populations of bacteria [41,42] – a phenomenon dubbed “roundabout selection” [42,43]. Figure 4 demonstrates the occurrence of roundabout selection in exemplary populations. Early in the runs (5,000 updates, at left), organisms evolving in the +EQU/-AND environment had much lower fitness in the +EQU environment than those that were evolving in the +EQU environment. However, by the end of the experiment (100,000 updates, at right), the same +EQU/-AND evolved population had achieved higher fitness in the +EQU environment than the organisms that evolved in that environment.

The finding that the +EQU/-AND populations, which started from the same ancestor but evolved in a different environment, had significantly higher fitness in the +EQU environment than the populations that evolved there strongly suggests the existence of multiple adaptive peaks. More precisely, it demonstrates that higher fitness is possible in the +EQU environment, and thus something must have prevented the +EQU-evolved populations from reaching that higher fitness. Nevertheless, although both EQU performance and fitness differed on average between the populations evolved under the two treatments, there was substantial variation among populations within each treatment. Thus, the results do not mean that populations in the +EQU environment always failed to reach the higher fitness peak, but only that they did not reach it as often as the populations that evolved in the +EQU/-AND environment.

**Figure 5:**
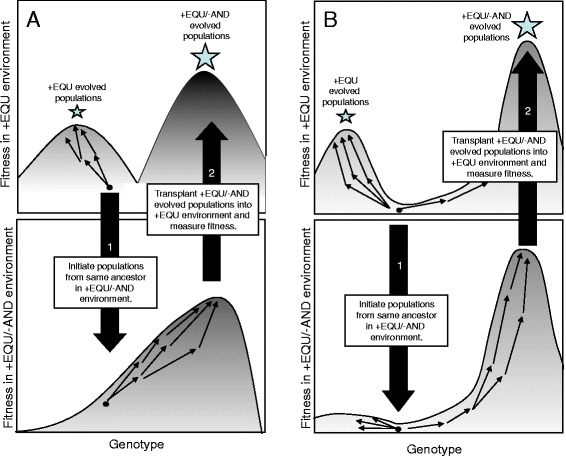
Two hypotheses about how ruggedness of the adaptive landscape might yield the observed results. Schematic shows two hypotheses that could explain the counterintuitive result in which populations that evolved in the +EQU/-AND environment reached higher fitness in the +EQU environment than did those that evolved in the +EQU environment. **(A)** Populations evolved to the lower fitness peak in the +EQU environment (upper left) because an adaptive valley prevented them from finding the higher peak. However, that valley did not exist in the +EQU/-AND environment (lower left), allowing selection to drive the populations up the single peak. When organisms that evolved in the +EQU/-AND environment were assayed for their fitness in the +EQU environment, they were on the higher peak. **(B)** The ancestor was between two adaptive peaks, either of which could be reached from that point. However, evolution in the +EQU environment (upper right) predisposed populations toward the lower peak because its initial ascent was steeper or mutations were more likely to move the populations into its domain of attraction. By contrast, evolution in the +EQU/-AND environment (lower right) predisposed populations to evolve toward the higher peak.

It is unclear what prevents the +EQU-evolved populations from reaching higher fitness. We see two related possibilities. First, the punishment for performing the AND function in the +EQU/-AND environment might alter the adaptive landscape in such a way that what was previously an adaptive valley becomes flat or even uphill from a now-sunken starting point (Figure 5A). In this case, the +EQU/-AND populations could have moved into areas of genotypic space that would have been less accessible in the +EQU environment owing to the intervening valley. When examined back in the +EQU environment, these +EQU/-AND populations would occupy a higher peak (Figure 5A). This process, whereby selection in a fluctuating environment permits populations to attain a higher fitness than would otherwise be possible, was described by Wright as “mass selection under changing conditions” [44,45].

A second possibility is that the ancestor sits at some distance from both peaks, and that either peak can potentially be reached from that starting point without crossing a valley (Figure 5B). However, in the +EQU environment, selection preferentially moves populations toward the lower peak, either because the initial ascent is steeper or because there are more paths leading to this peak than to the other. For example, progress toward the higher peak might require traversing a single narrow ridge, whereas there are many paths that lead to the lower peak. The key distinction between this hypothesis and the previous one is that there need not be an intervening adaptive valley that prevents the populations from finding the higher peak. Rather, the evolutionary trajectory might depend on the likelihood of populations stumbling upon the rare genetic variants that permit them to travel along the narrow ridge to the higher peak. Moreover, selection against AND in the +EQU/-AND environment would favor the loss of AND, perhaps increasing the likelihood of discovering the trajectory to this higher peak in the +EQU/-AND environment.

These hypotheses are not mutually exclusive; the correct explanation for the difference in the evolutionary trajectories might involve a mix of these and other processes. Nevertheless, support for the first hypothesis would involve showing that the trajectories of populations in the +EQU/-AND environment involved genotypic intermediates that would have been deleterious had they arisen in the +EQU environment, but which were neutral or even beneficial in the environment where they arose. Evidence for the second hypothesis would require finding that evolution in the +EQU/-AND environment often involved the substitution of the same few mutations in the replicate populations, which would support the idea that there was a paucity of paths that lead to the higher peak. Below, we present evidence that bears on these two possibilities.

### Fitness effects of mutations that resulted in the loss of AND

The only difference between the +EQU and +EQU/-AND environments was that performing AND reduced fitness in the latter. Thus, a good way to find mutations with differential fitness effects in the two environments would be to identify the mutations that caused the loss of AND in the +EQU/-AND environment. Forty-seven of the 100 populations that evolved in this environment lost AND (Table 3). This fact suggests that the adaptive-valley hypothesis is unlikely to explain fully the results, unless evolution in the +EQU/-AND environment reduced, but did not eliminate, the valley. Otherwise, we would expect the loss of AND in the +EQU/-AND environment to have occurred more often than it did or even invariably, as we saw in the five other cases we analyzed (Table 3, far right column). In any case, we determined the fitness effects of the mutational steps that caused the loss of AND in these 47 populations. In three populations, organisms along the line of descent lost AND, regained it, and then lost it again. For these three cases, we count only the first loss. In one other population, the genotype that first lost the AND function was not assigned a fitness by the test CPU (see Methods), so we excluded this case from our analysis. In the remaining 46 populations, the mutations that caused the loss of AND were universally beneficial in the +EQU/-AND environment where they arose. By contrast, 32 of these 46 mutational steps were deleterious when assayed in the +EQU environment, a further nine were neutral, and only five were beneficial. (The results are similar if we include instead the second, final loss of AND for the three populations that lost AND twice; the only difference is that one beneficial step is shifted into the neutral category.) Taken together, these data indicate that the AND function was often retained in the +EQU environment, at least in part, because its loss was usually caused by deleterious mutations. Changing the environment by imposing selection against AND thus opened up certain evolutionary paths that were not otherwise adaptive. This result provides clear support for our first hypothesis—that changing the environment altered the adaptive landscape in such a way that it allowed populations to evolve into regions that were otherwise inaccessible, enabling them to approach a peak of higher fitness.

### The number of paths leading to the loss of AND

**Figure 6:**
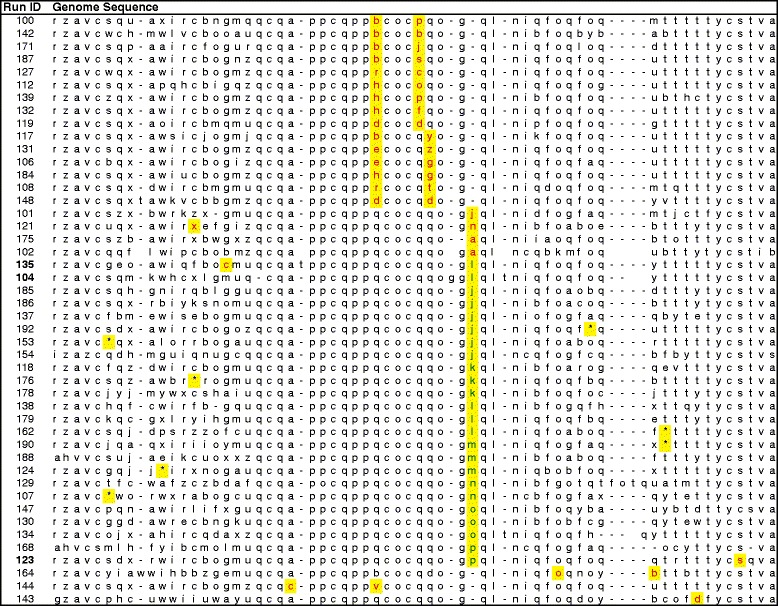
Genome alignment showing mutations associated with loss of the AND function. Aligned genome sequences of genotypes on the lines of descent in 46 populations, showing the mutations that were associated with the loss of the ability to perform the AND function. The immediate parent of each genotype could perform AND; thus, one or more of the mutations (highlighted in yellow) caused the loss of that ability. Each letter denotes a particular instruction; a red letter indicates a point mutation, whereas green letters and blue asterisks are insertion and deletion mutations, respectively. In three cases (bold run ID), the AND function was lost and regained multiple times, and only the first loss is shown. Several sequences have been trimmed owing to their length, and they show only the relevant portions of the genomes.

Our second hypothesis considered the relative likelihood of reaching one peak versus another owing to limitations on the production of relevant variation. To address it, we again focused on the mutations that caused the loss of the AND function. We examined the line of descent in the 46 populations that lost AND when it was selected against. In each case, we identified the particular genotype on this line that first lost the function. Figure 6 shows the alignment of the genome sequences of these genotypes; the mutations that distinguish each of these genotypes from its immediate parent are highlighted.

Several patterns are immediately apparent. First, either one or two of the same four sites changed in 44 of the 46 replicate populations. In many instances, replicate populations even converged on the exact same substitution. Second, and even more surprisingly, in 27 (59%) cases, the loss of AND was associated with a double mutation. Given the fact that the genomic mutation rate was 0.1 in these experiments, there should be, on average, 0.1 mutations per genome per generation. Because a genotype on the line of descent necessarily differs from its parent by at least one mutation, we calculated the probability that a particular genotype would differ from its parent genotype by two or more mutations. From the Poisson distribution, only about 4.9% of the genotypes with at least one mutation are expected to have two or more mutations. Thus, the mutations that caused the loss of the AND function involved double mutations almost 12 times more often than expected by chance. In fact, two pairs of sites were involved in 15 of the 27 double mutations (first 15 rows in Figure 6). These results argue strongly that the number of ways of generating this beneficial phenotype – which required eliminating the AND function while retaining the EQU function and all other aspects of organismal performance – were very limited. Importantly, the genotype-phenotype map (Figure 1) shows that there were many ways to knock out the AND function; therefore, it must have been the pleiotropic effects of most such mutations on EQU or other fitness components that placed such severe constraints on the mutations that could be substituted in the +EQU/-AND environment.

Finally, the overrepresentation of double mutations among those genotypes that lost the AND function is interesting its own right, because it implies that the component mutations were not beneficial when they arose individually in the +EQU/-AND environment. It supports the presence of an adaptive valley in a relevant part of the genotypic space in the +EQU/-AND environment, albeit a narrow valley that could be traversed by double mutations. This result may also explain why only some of the populations that evolved in this environment lost the AND function, despite selection for that loss. Although punishing the performance of AND caused its loss far more often than when it was not punished (Table 3), the mutations necessary to produce this loss while also retaining other critical functions were evidently so infrequent that many populations failed to lose it. Thus, these results demonstrate that constraints on the types of variation available in these populations limited their ability to reach the alternative, higher adaptive peak.

## Discussion

We used self-replicating computer programs, or digital organisms, to study the evolution of pairs of traits, or functions, that are genetically integrated, such that selection on one function often led to correlated changes in the other function. We also used selection against the performance of the second function, or punishment, to investigate whether, and how easily, pairs of integrated functions could be disentangled. Our results showed that correlated responses of genetically integrated functions were often highly asymmetric. These asymmetries were consistent with the ways in which these functions were encoded in the genome. In particular, the genomic sites that encoded one function were necessary for performing the other function, but the overlap was not complete. Rather, the instructions encoding one function (in this study, OR or AND) were a subset of those required for the other function (here, EQU). This asymmetry in the encoding of these functions was reflected in the asymmetry of their correlated responses to selection.

This form of pleiotropy, where multiple traits have a shared underlying genetic basis, can potentially limit adaptation in both biological and digital organisms. Moreover, the asymmetry of the correlated responses observed here – caused by the genes necessary for one trait being a subset of those required for another – is likely to be relevant to biological organisms as well, because that relationship arises whenever complex new functions are built on top of simpler ones [22]. Asymmetric correlated responses to selection are commonly observed in selection experiments [46-48] but, to our knowledge, asymmetries in the genetic encoding of the underlying traits has not been considered as a potential explanation. Nevertheless, while pleiotropy is often discussed as a potential constraint on adaptation, it can also play a positive role. For example, pleiotropy can maintain functions in environments where they are not under selection and would thus be subject to destruction by mutation accumulation [19,49]. Pleiotropy can also help maintain costly traits, such as cooperation, when the costly trait is functionally connected to another trait under strong positive selection [50,51].

The functions that showed correlated increases in response to selection for performing EQU were usually lost when they were selected against by punishing their performance. Thus, even traits that are tightly coupled can often be uncoupled by strong selection that favors their disassociation. Other studies that have examined selection on correlated traits have also shown that these associations can be modified by selection. For example, Lenski [45] showed that the fitness cost associated with a phage-resistance allele in *Escherichia coli* could be reduced by substituting modifier alleles that compensated for pleiotropic effects. In experiments with the butterfly *Bicyclus anynana*, Beldade et al. [34] showed that the relative size of anterior and posterior eyespots on their forewings could be uncoupled, albeit with some difficulty, while in that same species Zijlstra et al. [35] found that selection readily uncoupled the correlation between development time and eyespot size. Delph et al. [33] used artificial selection to try to break the correlation between flower size in males and females in the sexually dimorphic plant *Silene latifolia*, with mixed success across replicate lines. The fact that strong genetic correlations can often be decoupled suggests that they might have been generated, in part, by previous selection favoring certain trait combinations, rather than resulting from evolutionarily inflexible internal constraints [33,52]. Wright proposed a similar idea, suggesting that useless or even slightly deleterious traits might be retained for long periods of time owing to their genetic integration with other traits under selection for retention. He also emphasized, however, that these unused traits might be rapidly lost during times of “reorganization” [53,54].

All of the otherwise positively correlated responses were completely reversed when one of the functions was punished, with one striking exception (Table 3). In that case, over half of the 100 replicate populations that evolved in the +EQU/−AND environment, where performing EQU was rewarded while AND was punished, failed to lose the AND function. Even more surprising, populations in the +EQU/-AND environment evolved higher performance of EQU than populations that evolved in the +EQU environment, where performing AND was not selected against. This difference also translated to higher overall fitness, such that populations that had evolved in the +EQU/−AND environment were significantly more fit in the +EQU environment than those populations that evolved in that environment. This finding strongly suggested the existence of multiple adaptive peaks in the +EQU environment, and it implied that the populations that evolved in the +EQU environment were somehow prevented from reaching the higher peak.

We examined two hypotheses that might explain the failure of the +EQU-evolved populations to reach the higher fitness peak achieved by the populations that evolved in the +EQU/−AND environment. The first hypothesis involved a difference in the adaptive landscape between the +EQU/−AND and the +EQU environments, one that allowed populations in the +EQU/−AND environment to cross what was an adaptive valley in the +EQU environment and thereby reach a peak of higher fitness. Consistent with this hypothesis, mutations that caused the loss of AND were usually beneficial in the +EQU/−AND environment where they arose, but the same mutations would have been deleterious had they arisen in the +EQU environment. The finding that these mutations were deleterious in the +EQU environment explains why so few populations in that environment lost the AND function. That is, the evolutionary trajectories of populations in the +EQU/−AND environment often progressed through genotypic intermediates that would have been selected against – and hence inaccessible – in the +EQU environment.

Epistasis for fitness is required to generate rugged adaptive landscapes [8,44,55]. The work here and elsewhere indicates that such landscapes often emerge even in simple artificial-life systems [21,22,27]. Although there is considerable evidence that rugged adaptive landscapes also exist in nature, Wright’s Shifting Balance Theory, which incorporates a particular set of evolutionary forces to explain adaptation that occurs by moving between fitness peaks, remains controversial [11,12,56]. Part of the difficulty is that it is nearly impossible to determine in retrospect whether shifts between alternative adaptive peaks have occurred and, if so, by what mechanism. Our results support the idea of rugged, multi-peaked adaptive landscapes. However, the shifts we observed were not caused by genetic drift that allowed populations to cross an adaptive valley. In fact, we saw quite the opposite: 99 of the 100 populations that evolved in the +EQU environment never progressed toward the higher adaptive peak that involved the loss of the AND function. Only by changing the environment, such that movement toward the alternative peak became strongly beneficial, did we observe the peak shift. Thus, our results do not support Wright’s Shifting Balance Theory; instead, they support his alternative hypothesis that mass selection in a changing environment can promote peak shifts. Other studies using genetic algorithms have also shown that fluctuating environments can facilitate adaptation; not only can such fluctuations help populations avoid getting stuck on local fitness peaks, but they can sometimes lead to faster evolution [57].

We also considered a second hypothesis for the failure of most populations that evolved in the +EQU environment to find this alternative adaptive peak. The populations started at some distance from both peaks, and this hypothesis posits that the genetic coupling of the EQU and AND functions predisposed populations to evolve toward one or the other peak, depending on the environment. The importance of genetic correlations for movements on rugged adaptive landscapes was examined by Price et al. [37], who described how selection on a correlated trait could cause a population to shift between alternative adaptive peaks for some focal trait (see also [58]). Similarly, Schluter [38,59] showed how genetic correlations could lead to evolution along “genetic lines of least resistance.” The broader importance of these models is that they illustrate how the structure of genetic variation can influence the trajectory of a population evolving on a rugged adaptive landscape.

## Conclusions

Pleiotropy and epistasis are widely hypothesized to constrain or channel evolutionary change. However, empirical studies of their effects are challenging, and even strictly theoretical analyses are often hampered by the complexity of adaptive landscapes, which limits the scope of such analyses to a few loci or requires other simplifying assumptions [60]. We used populations of digital organisms to examine how pleiotropy and epistasis together affected the potential for evolution to independently optimize pairs of genetically integrated traits. While some trait pairs could be readily disentangled by selection, we found one case where the two traits could not be easily disentangled, such that one trait evolved counter to its phenotypic optimum owing to stronger selection on the other trait. By taking advantage of the transparency of the evolutionary process in digital organisms, we demonstrated how the shape of the underlying adaptive landscape influenced the evolutionary trajectories of these populations, leading to either the maintenance or loss of the genetically integrated traits. Our results thus shed new light on when and how pleiotropy and epistasis can constrain evolutionary outcomes, including their roles in promoting parallel or divergent evolutionary trajectories.

## Availability of supporting data

The datasets supporting the results presented in this article are available in the Dryad digital repository https://datadryad.org/resource/doi:10.5061/dryad.hh650.
